# Genome-Based Construction of the Metabolic Pathways of *Orientia tsutsugamushi* and Comparative Analysis within the Rickettsiales Order

**DOI:** 10.1155/2008/623145

**Published:** 2008-06-01

**Authors:** Chan-Ki Min, Jae-Seong Yang, Sanguk Kim, Myung-Sik Choi, Ik-Sang Kim, Nam-Hyuk Cho

**Affiliations:** ^1^Department of Microbiology and Immunology, College of Medicine and Institute of Endemic Diseases, Seoul National University Bundang Hospital and Medical Research Center, 28 Yongon-Dong, Chongno-Gu, Seoul 110-799, South Korea; ^2^Division of Molecular and Life Science, School of Interdisciplinary Bioscience and Bioengineering, Pohang University of Science and Technology, Pohang 790-784, South Korea

## Abstract

*Orientia tsutsugamushi*, the causative agent of scrub typhus, is an obligate intracellular bacterium that belongs to the order of Rickettsiales. Recently, we have reported that *O. tsutsugamushi* has a unique genomic structure, consisting of highly repetitive sequences, and suggested that it may provide valuable insight into the evolution of intracellular bacteria. Here, we have used genomic information to construct the major metabolic pathways of *O. tsutsugamushi* and performed a comparative analysis of the metabolic genes and pathways of *O. tsutsugamushi* with other members of the Rickettsiales order. While *O. tsutsugamushi* has the largest genome among the members of this order, mainly due to the presence of repeated sequences, its metabolic pathways have been highly streamlined. Overall, the metabolic pathways of *O. tsutsugamushi* were similar to *Rickettsia* but there were notable differences in several pathways including carbohydrate metabolism, the TCA cycle, and the synthesis of cell wall components as well as in the transport systems. Our results will provide a useful guide to the postgenomic analysis of *O. tsutsugamushi* and lead to a better understanding of the virulence and physiology of this intracellular pathogen.

## 1. Introduction

*O. tsutsugamushi*, an obligate intracellular bacterium, is the causative agent of scrub typhus [1] which is characterized by fever, rash, eschar, pneumonitis, meningitis, and disseminated intravascular coagulation that leads to severe multiorgan failure if untreated [2]. The mortality rate of scrub typhus in untreated patients ranges from 1 to 40%, depending on the patient condition, the endemic area, and the strain of *O. tsutsugamushi* [3]. Scrub typhus is confined to a geographical region that extends from far eastern Russia and northern Japan in the north, to northern Australia in the south, and Pakistan and Afghanistan in the west [3]. The principal ecologic feature that distinguishes scrub typhus from other enzootic rickettsiosis is related to the distribution and life cycle of trombiculid mite vectors and their vertebrate host [3]. Human infection by *O. tsutsugamushi* is mediated through the bites of the larva of the trombiculid mite, which harbor the bacterium in their salivary glands [4].

Although scrub typhus can be treated effectively with antibiotics such as doxycycline and chloramphenicol, reinfection and relapse frequently occur due to the wide variety of antigenically distinct serotypes [5]. Furthermore, decreased effectiveness of antibiotic treatments was recently reported in several cases [6, 7]. While the number of patients with scrub typhus and recurrent outbreaks has recently increased in endemic areas [6, 8, 9], an effective vaccine has yet to be developed, possibly due to the limited duration of the immune response [10] and immunosuppression in the infected host [11].

*Orientia* belongs to *α*-proteobacteria and was reclassified as a new genus distinct from *Rickettsia* based on phenotypic and genotypic differences [12]. *Orientia* differs from *Rickettsia* in the structure of the cell wall, antigenic profile, and genome size, which is almost twice the size of the *Rickettsia* genome [13]. We have recently completed sequencing of the genome of *O. tsutsugamushi*, and shown that it contains the highest content of repeated sequences (approx. 40% of the genome) among bacterial genomes sequenced to date. We also showed that the repeats are generated by the massive proliferation of mobile genetic elements such as conjugative type IV secretion system components and transposons [14].

The members of the Rickettiales order, which is divided into the Anaplasmataceae family (*Wolbachia*, *Anamplasma*, *Ehrlichia,* and *Neorickettsia*) and the Rickettsiaceae family (*Rickettsia* and *Orientia*) are associated with a diverse set of hosts and vectors which exhibit a range of mutualistic and parasitic relationships. Host switching and differences in the mode of transmission, from transovarian to horizontal transmission, create additional diversity in the host-parasite relationship [15]. Recently, the wealth of genomic information for the Rickettsiales members including the agents of scrub typhus, epidemic typhus, ehrlichioses, and heartwater disease has provided valuable resources for exploring the effect of host association on the evolution of intracellular pathogenic bacteria [14–19].

The characterization of the metabolic properties of intracellular bacteria as well as the mechanisms by which these pathogens acquire nutrients from their host, is important in understanding virulence and related diseases. Genome-based construction of the metabolic pathways of intracellular pathogens may provide valuable insights into their pathogenic properties as well as indicate potential targets for the development of novel therapeutics. In the current study, we have generated a detailed map of the metabolic pathways of *O. tsutsugamushi* based on genomic information, and compared the metabolic features of *O. tsutsugamushi* to other members of the Rickettsiales order.

## 2. Methods

Metabolic and genetic analysis of *O. tsutsugamushi* was based on previous published annotation data [14, 19]. Annotation of COGs of putative functional genes was further confirmed by performing a BLAST search against the COG database (e-value < 10^−10^, multiple assignments per protein allowed) [20]. Metabolic pathways were subsequently analyzed using the Kyoto encyclopedia of genes and genomes (KEGGs) metabolic database [21]. Each gene that was implicated in a metabolic pathway was manually confirmed by a BLAST search of KEGG genes using the web-based BLASTP program (e-value < 10^−20^). Genes encoding putative transporters were identified based on the TransportDB database [22] and KEGG membrane transport data. Annotated transporters of 9 Rickettsiales members in TransportDB were collected and used to identify homologous transporters in *Orientia*. Additional putative transporters that were previously annotated [14] were analyzed using the web-based BLASTP program (e-value < 10^−20^). Among 1249 genes of *O. tsutsugamushi*, which excluded the genes for mobile genetic elements, 819 genes were annotated with functional COGs and used for the metabolic construction. Ortholog searches (length ratio criteria 80% and cutoff e-value < 10^−10^) within the members of Rickettsiales were performed by BLASTP searches using formatted data collected from the genomes of *Rickettsia conorii* (NC003103), *R. bellii* (NC007940), *R. felis* (NC007109), *R. typhi* (NC006142), *R. prowazekii* (NC000963), *Wolbachia* endosymbiont strain TRF of *Brugia malayi* (NC006833), *Wolbachia* endosymbiont of *Drosophila melanogaster* (NC002978), *Anaplasma marginale* (NC004824), *A. phagocytophilum* (NC007797), *Ehrlichia canis* (NC007354), *E. ruminantium* (NC006832, NC005295), *E. chaffeensis* (NC007799), *Neorickettsia sennetsu* (NC007798), and *O. tsutsugamushi* (NC 009488).

## 3. Results and Discussion

### 3.1. Carbohydrate and Energy Metabolism

**Figure 1:**
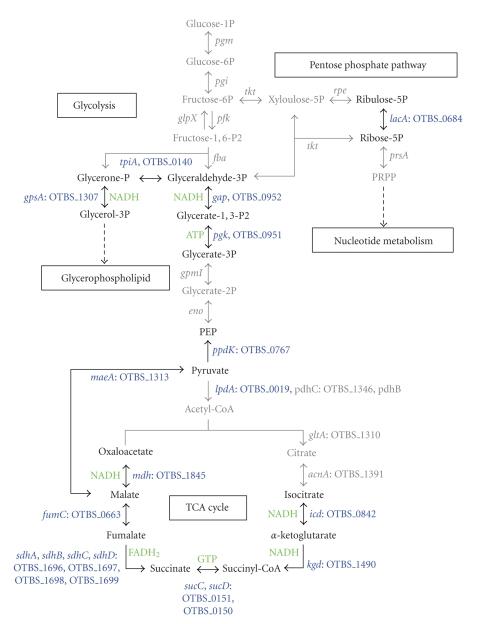
Glycolysis and the TCA cycle in *O. tsutsugamushi*. Missing components and the resultant metabolites that are not synthesized are indicated in gray. Steps that generate reductive power or lead to the formation of ATP by substrate-level phosphorylation are indicated in green. The genes and corresponding CDSs present in the genome are indicated in blue, and pseudogenes and genes that are absent are shown in gray.

Glycolysis and the citric acid cycle are the major energy-producing catabolic pathways and they are conserved in all kingdoms of life. Genome sequence analysis revealed that some of the enzymes of these pathways are present in *O. tsutsugamushi*. Three enzymes of the glycolysis pathway, in which glucose is oxidized to pyruvate, were present (*gap*, *pgk*, and *tpiA*; Figure 1). These enzymes may be used to generate glycerol phosphate which is the starting material for the synthesis of glycerophospholipids or used to generate energy from glycerol-3-phosphate by a reverse reaction. *Rickettsia* do not possess these enzymes [23], but they possess a gene for glycerol phosphate transporter (*glpT*) which imports the material from host cells (Table 1 and Figure 7). A limited number of enzymes of glycolysis were identified in the genomes of *Wolbachia*, *Anaplasma*, and *Ehrlichia,* and it has been suggested that in these organisms the synthesis of glyceraldehydes-3-phosphate may be the mechanism used for the production of pentose, a cofactor that is required for nucleotide biosynthesis [17]. The presence of the gene for fructose bisphosphatase (*glpX*), which is the key enzyme of gluconeogenesis, in these organisms provides further evidence that the enzymes of the glycolysis pathway are used in gluconeogenesis rather than glycolysis (Figures 1 and 8). Consistent with the limited capability for carbohydrate metabolism, the genes for sugar phosphate transporters or hexokinases were barely identified in all of the members of Rickettsiales, which suggests that most of the energy in the members of this order is obtained from amino acids, rather than hexose catabolism (see below). Pyruvate is the major product of glycolysis and is used in multiple metabolic pathways. In *O. tsutsugamushi*, pyruvate would not be synthesized by the glycolytic pathway (Figure 1). It is possible that it is acquired from the host, or synthesized from malate by malate dehydrogenase (*maeA*), and then subsequently converted to phosphoenol-pyruvate by pyruvate phosphate dikinase (*ppdK*), both of which are present in all of the Rickettsiales members (Figures 1 and 8). The presence of a putative permease (OTBS_1312, Table 1) that is found only in *Rickettsia* and *Orientia* and may function in transporting malate from the host cell [24] further supports this idea.

 It seems likely that pyruvate does not fuel the citrate acid cycle in *O. tsutsugamushi* because the three enzymes involved in the initial steps of the pathway are lacking [14, 19]. Among the members of Rickettsiales, *O. tsutsugamushi* is the only member that lacks the functional pyruvate dehydrogenase complex and has to rely on the host cell as a source of acetyl-CoA, which is an essential coenzyme in diverse biosynthetic pathways. The only functional component of the pyruvate dehydrogenase complex that we identified was dihydrolipoamide dehydrogenase (*lpdA*), whereas the other two subunits, *pdhB* and *pdhC,* were absent or present as a pseudogene. *gltA* and *acnA* which encode citrate synthase and aconitate hydratase, respectively, are also present as pseudogenes in *O. tsutsugamushi*, while most of the components of the energy-yielding reactions between isocitrate dehydrogenase (*icd*) and malate dehydrogenase (*mdh*) are present [14, 19]. Considering the lack of the initiating enzymes of the citric acid cycle, this pathway in *O. tsutsugamushi* would start with *α*-ketoglutarate and end with oxaloacetate. Consistent with the presence of a glutamate transport system (GltP), the putative glutamine ATP-binding cassette (ABC) transporter (*ygiX*, *ygiY*, and *glnQ*), and glutamine synthase (*glnA*), it is possible that glutamate is imported from the host cell or synthesized from glutamine by GlnA (Figures 3, 7, and Table 1). Glutamine is converted to aspartate and *α*-ketoglutarate by AatA, using oxaloacetate as a cosubstrate and could fuel the “minimal” citrate acid cycle to generate energy [19].

 ATP is the universal energy source in all biological systems, and can be synthesized by glycolysis and oxidative phosphorylation. As in other *Rickettsia* [23], *O. tsutsugamushi* possesses the majority of genes for oxidative phosphorylation including three proton pumps, the succinate dehydrogenase complex, and the ATP synthase complex. Another mechanism of acquiring ATP is to import host ATP through ATP/ADP translocases which are present in *Rickettsia* and *Chlamydia* [25]. *O. tsutsugamushi* has five copies of the ATP/ADP translocase (Figure 7 and Table 1). These obligate intracellular pathogens may first exploit ATP that is already present in the host cell cytoplasm through the function of translocases and subsequently produce ATP via aerobic respiration when the host pool of ATP has been consumed [23]. Recently, it was demonstrated that the translocases of *Rickettsia* have differential transportation properties for nucleotides [26]. In addition to exchanging bacterial ADP for host cell ATP as a source of energy, some of the translocases are believed to function primarily in maintaining intracellular pools of nucleotides for rickettsial nucleic acid biosynthesis. Consistent with this, *Rickettsia* and *Orientia* are generally deficient in the enzymes of de novo nucleotide synthetic pathways.

### 3.2. Nucleotide Metabolism

**Figure 2:**
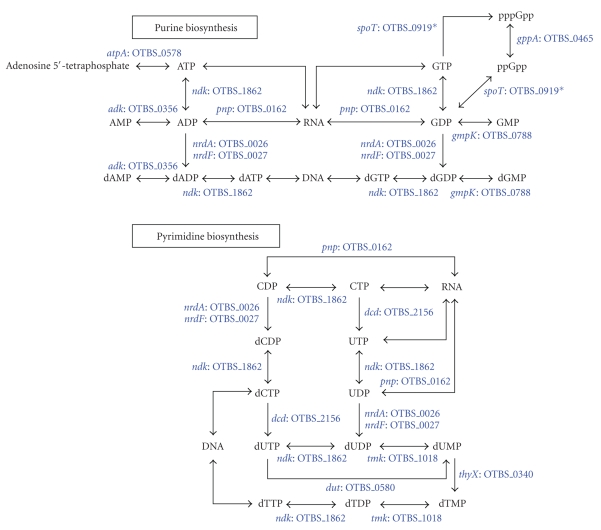
Nucleotide biosynthesis. Components of purine and pyrimidine metabolism that are present in the genome. As described in the text, it is likely that only the salvage pathways are functional.

Several components of the salvage pathways of purine and pyrimidine biosynthesis were present in *O. tsutsugamushi*, similar to other *Rickettsia* (Figure 2) [23]. The absence of enzymes for the interconversion of adenine and guanine suggests that these bacteria depend on the host for the both purines and may import them via different subtypes of ATP/ADP translocases [26]. In contrast, the interconversion of pyrimidine nucleotides is feasible in *O. tsutsugamushi* due to the presence of deoxycytidine triphosphate deaminase (*dcd*) and FAD-depedant thymidylated synthase (*thyX*) (Figure 2). The gene (*codA,* OTBS_1716) for cytosine deaminase, which converts cytosine into uracil, is present in all the members of Rickettsiales.

Phosphorybosyl pyrophosphate (PRPP), which is produced by the pentose phosphate pathway, is a key metabolite in the synthesis of nucleotides. *O. tsutsugamushi* lacked all the enzymes for this pathway with the exception of ribose-5-phosphate isomerase (LacA) (Figure 1). A similar deficiency in the enzymes of this pathway was reported for *Rickettsia* [23], while the gene for ribose-phosphate diphosphokinase (*prsA*) has been identified in *R. felis* and *R. bellii*. These bacteria may synthesize PRPP from ribulose-5-phosphate which may be imported from the host cell. In contrast to *Orientia* and *Rickettsia*, the members of Anaplasmataceae are well equipped with the enzymes for the de novo nucleotide synthetic pathways and the pentose phosphate pathway (Figure 8).

The guanosine nucleotides pppGpp and ppGpp are important second messengers in the bacterial stringent response to cope with nutritional starvation [27]. *O. tsutsugamushi* is the only bacterium of the Rickettsiales order that encodes a fully bifunctional *spoT/relA* homologue [14, 15, 19]. Even though *Orientia* and some *Rickettsia* have several short ORFs encoding either the hydrolase or synthase domains, the biological significance of these is unknown [28].

### 3.3. Amino Acid Metabolism

**Figure 3:**
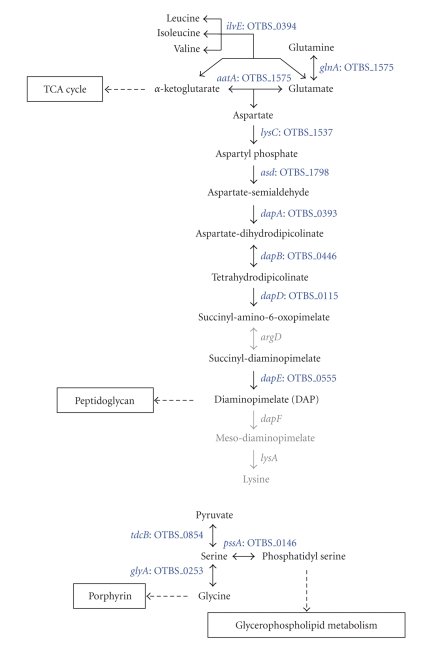
Amino acid metabolism. The components that are absent from the genome are indicated in gray. Lysine biosynthesis is partially present and may provide DAP for peptidoglycan synthesis.

 Amino acid metabolism appeared to be limited in *O. tsutsugamushi* (Figure 3). A similar level of deficiency in the amino acid metabolic pathways of *Rickettsia* has also been described [23]. The genes for converting glutamine to glutamate (*glnA*), glutamate to *α*-ketoglutarate and aspartate (*aatA*), serine to glycine (*glyA*), and branched-chain amino acid aminotransferase (*ilvE*) were present in the genome of *O. tsutsugamushi*. In contrast, the members of Anaplasmataceae, particularly *Ehrlichia*, have a greater capacity to synthesize amino acids such as proline, arginine, and lysine (Figure 8) [17]. Three genes that are involved in the biosynthesis of lysine from aspartate, *argD*, *dapF,* and *lysA*, were missing in *O. tsutsugamushi*. Among the Rickettsiales, *argD* is absent in *Orientia* and *Rickettsia*, while *dapF* is missing only in *Orientia*. *lysA*, which encodes diaminopimelate decarboxylase, is present only in the genus *Ehrlichia*. Given that these genes are not present in *Orientia*, they may be involved in the synthesis of diaminopimelate, which is an important component of peptidoglycan, rather than lysine biosynthesis. Genes for the biosynthetic pathways for aromatic amino acids (tryptophan, tyrosine, and phenylalanine), and for histidine are missing in *Orientia* as well as other *Rickettsia* species. Those amino acids must be provided by the host cell or the culture medium. In contrast to *Rickettsia*, *Orientia* lacks the alanine racemase (*alr*) which converts L-alanine to D-alanine, a key component of peptidoglycan [19]. However, it has the enzymes (*ddl*, *murD*, and *murF*) for incorporating D variants of amino acids into peptidoglycan, which suggests that this bacterium may obtain D-amino acids from the host cell, or use the L variants for murein biosynthesis [29]. For aminoacyl-tRNA synthesis, it is notable that two copies of tryptophanyl-tRNA synthetase and two pseudogenes for phenylalanyl-tRNA synthetase alpha chain, in addition to all 20 aminoacyl tRNA synthetases, were present in the genome of *O. tsutsugamushi*.

### 3.4. Lipid Metabolism and Cell Wall Structure

**Figure 4:**
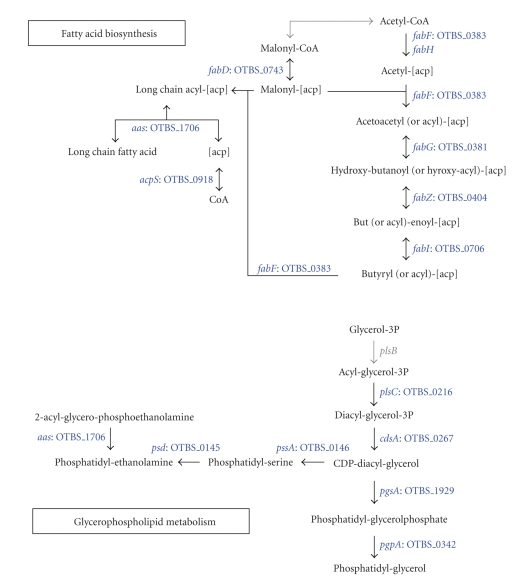
Phospholipid synthesis. The pathways for the interconversion of phosphatidyl glycerol, phosphatidyl serine, and phosphatidyl enthanolamine are complete as well as the pathway for fatty acid biosynthesis.

 The majority of genes for fatty acid biosynthesis were present in *O. tsutsugamushi*, as in other members of the Rickettsiales order (Figure 4). An important exception was *fabH* which encodes beta-ketoacyl-acyl carrier protein (ACP) synthase III of the fatty acid elongation reaction. Interestingly, activation of long chain fatty acids with ACP by acyl-ACP synthetase (*aas*) may occur in *Orientia* and *Rickettsia*. The *aas* gene product catalyzes the synthesis of acyl-ACP from a long chain fatty acid which may be imported from the host cell, and activated acyl-ACP might subsequently play a role in the incorporation of fatty acids into phospholipids [30]. The entire set of genes for phospholipid synthesis (*plsC*, *cdsA*, *pssA*, *psd*, *pgsA*, and *pgpA*) with the exception of *plsB* which encodes an enzyme involved in the incorporation of the first acyl chain into glycerol-3-phosphate, are present in all the members of Rickettsiales (Figure 4). The *β*-oxidation system of fatty acids for energy generation was absent in *Orientia,* whereas this pathway is present in *Rickettsia* [23].

One of the major constituents of the outer cell membrane in Gram-negative bacteria is lipopolysaccharide (LPS). Genomic analysis of *O. tsutsugamushi* indicated that it lacks the genes for the biosynthesis of lipid A, as suggested by previous biochemical analyses [31]. Among the members of Rickettsiales, only *Rickettsia* are equipped with the genes required for lipid A biosynthesis (Figure 8) [19]. Consistent with this, the genes for the O-antigen export system (*rfbA, rfbE*) are present only in *Rickettsia*.

**Figure 5:**
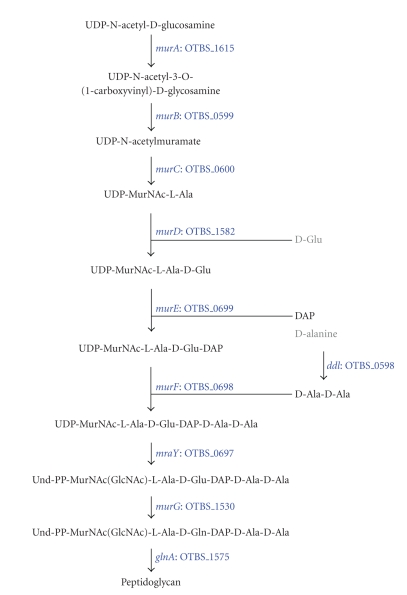
Peptidoglycan biosynthesis. The pathway for the synthesis of peptidoglycan from UDP-N-acetyl-D-glucosamine is complete. Even though the D-amino acids such as D-glutamate and D-alanine are not likely to be synthesized by *O. tsutsugamushi*, the genes for incorporating them into glycan are present.

The genes for cell wall biosynthesis were present in the genome of *O. tsutsugamushi* (Figure 5), similar to *Rickettsia* and *Wolbachia.* Interestingly, *A. marginale* also has the same set of genes for peptidoglycan synthesis with the exception of *murC* which is present as a pseudogene [32]. Other species of *Anaplasma* and *Ehrlichia* completely lack these enzymes (Figure 8). Furthermore, the complete aminosugar metabolic pathway for the synthesis of UDP-N-acetyl muramate which is an initiating material for peptidoglycan biosynthesis from fructose-6-phosphate is present (*glmS*, *glmM*, *glmU*, *murA*, and *murB*) in *A. marginale*, whereas *glmS*, *glmM*, and *glmU* are missing in *O. tsutsugamushi*. Penicillin-binding proteins are involved in the last stages of peptidoglycan biosynthesis, and mediate the transglycosylation and transpeptidation reactions [33]. Two penicillin-binding proteins (OTBS_0700 and OTBS_2173) that were identified in *O. tsutsugamushi* are conserved in *Rickettsia*, *Wolbachia*, and *A. marginale*. Another penicillin-binding protein, *pbpE*, is found in *Orientia* (OTBS_0205) and *Rickettsia*. *AmpC*, a *β*-lactamase which regulates cell shape [33], is present only in *R. felis* and *R. conorii*.

Given the differential distribution of genes for cell wall components in the genomes of arthropod- and mammalian-associated pathogens, it is possible that these bacteria may have evolved to modify their envelope structures depending on the specificity of their host where they have adapted. The components of the bacterial envelope, such as LPS and peptidoglycan, are strong inducers of the innate immune responses through Toll-like receptors, which are conserved from insects and mammals [34]. During the course of intracellular adaptation in higher eukaryotes, the presence of immune-stimulating components in the bacterial envelope may have been critical for a successful association with the host. The differential loss of genes for envelope biogenesis has also occurred during the evolution of the other insect-associated endosymbionts [29]. Although the common ancestor of Rickettsiales may have all the components of the cell wall as in other free-living *α*-proteobacteria, it might have gradually lost sets of genes for the macromolecular biosynthesis during the course of reductive genomic evolution and intracellular adaptation.

 The antigenic variability of *O. tsutsugamushi* among diverse strains is generated by several immunogenic proteins such as 110 kD, 56 kD, and 47 kD proteins, and it has hampered the development of a protective vaccine [1, 35]. The 56 kD strain-specific antigen, which is the main source of antigenic heterogeneity among different strains [1], is present as a single copy, and no homologous protein has been identified in other bacteria. Another major antigen, 47 kD group-specific protein, has been identified, and close homologues are found in diverse *α*-proteobacteria including all the members of Rickettsiales. The conserved 47 kDa antigen may not contribute to the antigenic variability of *Orientia*. We also identified two additional OmpA-like membrane proteins (OTBS_0601, OTBS_1984) which may contribute to the surface antigenicity of *O. tsutsugamushi*. It is interesting to note that one of the OmpA-like proteins (OTBS_0601) is located just downstream of the 56 kD protein, suggesting that these genes may form an operon and be expressed coordinately [14]. Like other *Rickettsia* species, *O. tsutsugamushi* has multiple types of Sca family genes (OTBS_0102, OTBS_0864, OTBS_1686, OTBS_1913, OTBS_2126, and OTBS_2137) containing autotransporter domains [36]. Several of these surface proteins in *Rickettsia* are known as antigenic determinants and may play a role in adhesion to host cells [37, 38]. Recently, It has been shown that Sca5 (OmpB) of *R. conorii* is involved in adhesion to host cells via membrane-associated Ku70 [38]. We previously identified 6 ORFs encoding genes containing autotransporter domains [14, 19]. None of them showed close homology with other autotransporters of *Rickettsia* outside the autotranspoter domain. Four of them have putative transmembrane domains in their N terminus. Two autotransporters that do not contain a transmembrane domain are encoded by the same gene (OTBS_0864 and OTBS_2137) which may have been duplicated through the action of a transposase. The presence of multiple types of autotransporters in *O. tsutsugamushi* may contribute to bacterial pathogenesis, in addition to surface antigenicity.

### 3.5. Metabolism of Cofactors

**Figure 6:**
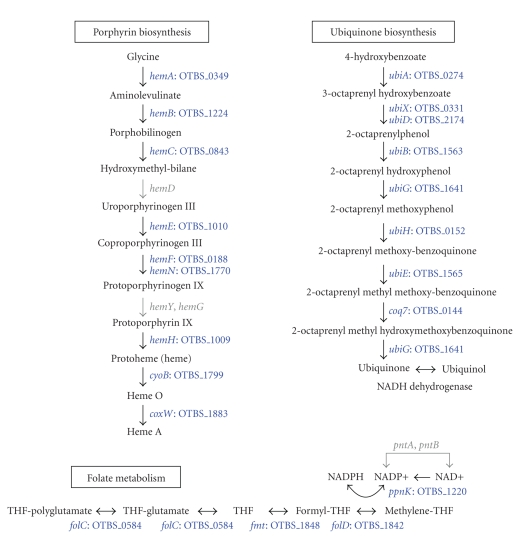
Metabolism of Cofactors. The biosynthetic pathways for porphyrin (heme) and ubiquinone are present in *O. tsutsugamushi*. Several genes for folate metabolism were also present in the genome.

The pathways for de novo biosynthesis of the vitamins and cofactors were generally absent in *O. tsutsugamushi*. The bacterium retained only parts of the biosynthetic pathways for heme (*hemA, hemB, hemC, hemE, hemF, hemH, ctaB*, and *cox15*) and ubiquinone (*ubiA, ubiD, ubiB, ubiG, ubiH, ubiE,* and *coq7*) (Figure 6). *hemD*, which encodes uroporphyrinogen-III synthase, is present as a pseudogene in *O. tsutsugamushi* (OTBS_1020) and *Rickettsia*. The gene for protoporphyrinogen oxidase (*hemY, hemG*) is absent in all the Rickettsiales members. A few genes for the final components of folate metabolism were also present in *O. tsutsugamushi* (Figure 6). The genes (*pntA* and *pntB*) for the NAD(P) transhydrogenase complex are present only *Rickettsia*.

 The presence of several essential vitamin and cofactor biosynthetic pathways in the members of Rickettsiales, particularly Anaplasmataceae, suggests that they do not compete with the host cell for these nutrients and may even supply host cells with essential vitamins and cofactors [17, 39, 40]. However, the ability to synthesize some cofactors is somewhat limited in *Wolbachia*, and even more so in *Rickettsia*, and *Orientia*. *W. pipientis* has completely lost the biosynthetic pathways for biotin, thiamine, and NAD. *R. prowazekii* has lost the ability to synthesize these cofactors as well, and in addition, cannot synthesize FAD, pantothenate, and pyridoxine-phosphate, which can be produced by *Anaplasma*, *Ehrlichia*, and *Neorickttsia* [17]. *O. tsutsugamushi* possesses the least capability for vitamin and cofactor synthesis among the sequenced Rickettsiales members.

### 3.6. Membrane Transporters

**Figure 7:**
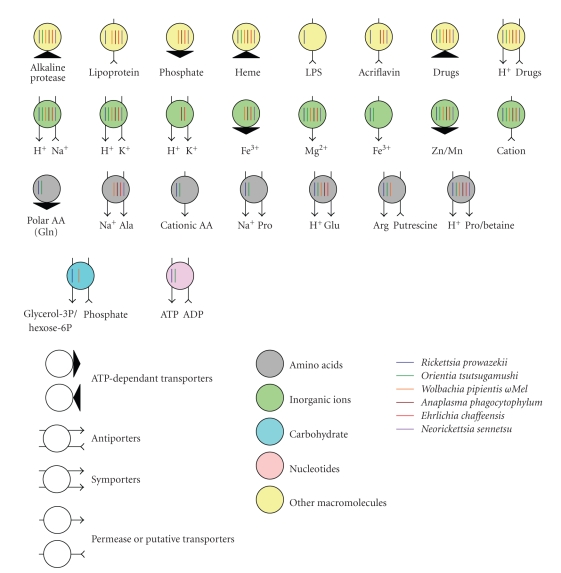
Comparative analysis of transporters and their putative substrates in five representative members of the Rickettsiales order. The types and putative substrates of the nutrient transporters are as indicated in the figure and legend box. The figures were constructed using data from transportDB (http://www.membranetransport.org/). The transporters of *O. tsutsugamushi* were predicted using a BLASTP search against the transporter database. It is notable that there are more transporters for amino acids and nucleotides in *Orientia* and *Rickettsia* than in members of Anaplasmataceae. The downward direction of the arrows indicates flow into the bacteria.

**Figure 8:**
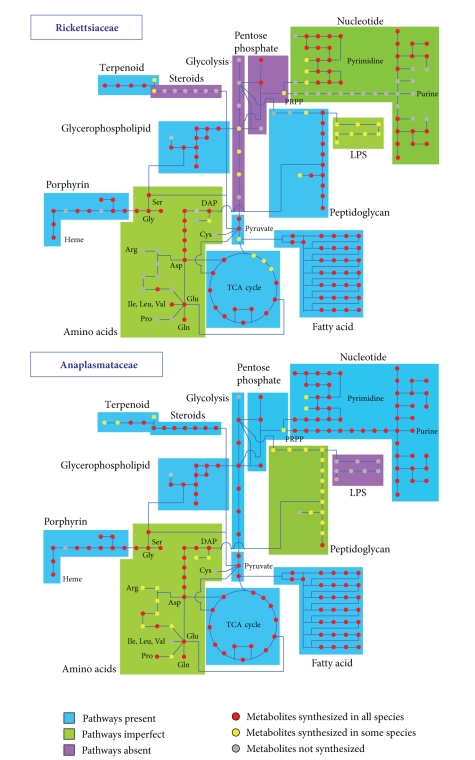
Comparison of the metabolic pathway maps of Rickettsiaceae and Anaplasmataceae. The overview of maps is color coded to indicate pathways that are complete, partial, or absent in Rickettsiaceae and Anaplasmataceae, based on the data from KEGG DB (http://www.genome.ad.jp/kegg/), as described in Section 2. The upper panel showing the metabolic map of Rickettsiaceae is slightly modified from the data reported by Fuxelius et al. [19].

 The transporter systems of *Rickettsia* consist mainly of secondary transporters, in which transport activity is driven by an ion gradient across the membrane and ABC-type transporters which are driven by ATP hydrolysis [23, 24]. While the substrates of these transporters in Rickettsiales members have not been well defined, putative substrates have been identified by amino acid sequence-based BLAST searches and domain predictions [24]. As shown in Table 1 and Figure 7, *O. tsutsugamushi* has a similar level of transporters as *Rickettsia* [23]. Both genuses have multiple types of amino acids transporters, as well as 4-5 copies of ATP/ADP transporters, which are absent in the members of Anaplasmataceae. Deficiencies in the pathways for amino acid biosynthesis in *Rickettsia* and *Orientia* could be partly overcome by the presence of multiple amino acid transporters (*proP,* *atrC1*, *panF*, and *potE*) (Figure 7 and Table 1). A putative sodium/alanine symporter (orthologs of AM882) is present exclusively in Anaplasmataceae family members (Figure 8). In addition, two ABC-type transporter systems for phosphate (*pstA*, *pstB*, *pstC*, and *pstS*) and iron (*afuA*, *afuB*, *afuC*) were missing only in *Orientia* and *Rickettsia*. A putative iron permease (OTBS_0219) may function as an iron transporter in *Orientia* and *Rickettsia*. Several transporters such as the lipoprotein export system (*lolC*, *lolE*, and *lolD*), lipid A transporter (*rfbA* and *rfbE*), and glycerol-3-phosphate transporter (*glpT*) were absent in *O. tsutsugamushi*, in contrast to other *Rickettsia* species. Protein secretion systems such as the Sec-mediated translocation system and the type IV secretion system, other than the highly amplified conjugative forms are conserved in all the members of Rickettsiales including *Orientia*.

## 4. Conclusion

We performed an analysis of the genomic sequence of *O. tsutsugamushi* and have identified several fundamental properties of the bacterium including metabolic properties and cell wall structure. Genome-based metabolic construction of *O. tsutsugamushi* revealed that this organism has limited central metabolic and biosynthetic capability, similar to other *Rickettsia* species. Even though it has the largest genome among the members of Rickettsiales, it lacks a majority of the components of the major biosynthetic pathways. Rather, it has streamlined its metabolic pathways to a greater extent than other *Rickettsia*. Due to its limited metabolic capabilities, *O. tsutsugamushi* most likely relies on its host for many organic nutrients. In this regard, the presence of a diverse set of transporters for various nutrients that is required for bacterial growth within the host cell may compensate for the lack of metabolic pathways (Table 1 and Figure 7). In light of its apparently extreme dependence on the host cell for nutrients, the bacterium may have evolved to retain sensors that react to environmental changes, and to regulate growth within the host cell. This may be particularly true for arthropod hosts, in which the bacterium has evolved for several hundreds of millions of years [15]. *O. tsutsugamushi* encodes a fully functional stringent response regulator, *spoT/relA*, multiple types of two-component signal transduction systems, and diverse host interaction genes, all of which may be involved in the bacteria-host interaction [14]. Modification of the bacterial envelope and the presence of multiple membrane proteins with antigenic potential indicate that the bacterium may have been evolved under pressure from the host immune system [41].

In addition to gaining a better understanding of the basic physiology and virulence of intracellular pathogens, the development of improved diagnostics, vaccines, and novel therapeutics is one of the major aims of bacterial genome analysis. Postgenomic approaches such as expression profiling during growth within a mammalian host cells have to be undertaken in order to achieve these ultimate goals. In addition, functional studies of putative proteins of unknown function, which comprise approximately 30% of the total CDSs of the *O. tsutsugamushi* genome, must also be carried out and may provide additional therapeutic targets. In particular, potential cell surface proteins and host interaction proteins could be placed as genes of priority in future functional studies.

## Figures and Tables

**Table 1:** Membrane transport systems found in the *O. tsutsugamushi* genome.

Transporter type	Gene/CDS			Putative substrate
ATP dependant (ABC family)	ABC	Membrane	Binding protein	
			*yqiX*: OTBS_0447	Amino acid
		*yqiY*: OTBS_0224		Glutamine
	*glnQ*: OTBS_0223			Glutamine
	*mkI*: OTBS_0496	*yrbE*: OTBS_0495	*yrbD*: OTBS_1584	Toluene tolerance
	*znuC*: OTBS_1910	*znuB*: OTBS_1909	*znuA*:OTBS_0441	Zinc, manganese
	*ccmA*: OTBS_1392	*ccmB*: OTBS_1393		Heme
		*ccmC*: OTBS_1577		Heme
		*msbA1*: OTBS_0552*		Multidrug
		*msbA2*: OTBS_0995*		Multidrug
		*aprD*: OTBS_0305*		Alkaliine protease
	*abcZ*: OTBS_0269			
		*abcT3*: OTBS_0695*		
	*abcT1*: OTBS_0781			

Ion channels	OTBS_1945			Mechanosensitive ion

Secondary transporter	*tlc*: OTBS_0312, OTBS_0313,			ATP/ADP antiporter
	OTBS_0547, OTBS_1035, OTBS_1636			
	OTBS_1312, OTBS_0017?			Malate
	*atrC1*: OTBS_1417			Cationic aminio acid
	*potE*: OTBS_1403			Arginine/orinithine
	*p34*: OTBS_1715			Cation
	*kefB*: OTBS_1539			Potassium
	*gltP*: OTBS_0443			Proton/glutamate
	*rarD*: OTBS_0203, OTBS_0407?			S-adenosylmethionine
	*proP*: OTBS_0158, OTBS_0204,			Proline/betaine
	* * * *OTBS_0844, OTBS_1493, OTBS_1955			
	*panF*: OTBS_0515, OTBS_1379,			Na^+^/proline symporter
	*ampG*: OTBS_0516			Muropeptide
	*bcr*: OTBS_1499, OTBS_1891			Multidurg
	*mnhE*: OTBS_0147, mnhG: OTBS_1421,			Na^+^/H^+^ antiporter
	mbhE: OTBS_1422			
	*mnhB*: OTBS_1423, mnhC: OTBS_1424			(multisubunit antiporter)

Unclassified	*mgtE*: OTBS_0470			Magnesium
	OTBS_0219?			iron

*Contains both an ABC and a membrane domain as one polypeptide.
